# Pterostilbene Reduces Cyclophosphamide-Induced Interstitial Cystitis by Facilitating Nrf2 Activation and Suppressing the NLRP3 Inflammasome Pathway

**DOI:** 10.3390/ijms26125490

**Published:** 2025-06-08

**Authors:** Jiong Zhang, Jipeng Wang, Xinhao Wang, Zehao Yan, Lingfeng Meng, Yaoguang Zhang

**Affiliations:** 1 Peking University Fifth School of Clinical Medicine, Beijing 100730, China; jiong_zhang1997@163.com (J.Z.); yzh2073104129@163.com (Z.Y.); 2 Beijing Hospital, National Center of Gerontology, Institute of Geriatric Medicine, Beijing 100730, China; wjp19940219@126.com (J.W.); wxh550@hsc.pku.edu.cn (X.W.); menglfdzs@163.com (L.M.)

**Keywords:** interstitial cystitis/bladder pain syndrome (IC/BPS), pterostilbene, inflammatory response, NLRP3 inflammasome, oxidative stress

## Abstract

Interstitial cystitis/bladder pain syndrome (IC/BPS) causes significant discomfort in patients and impairs the quality of urination. Pterostilbene (PTE), a natural polyphenol antioxidant, has demonstrated beneficial effects in mitigating inflammation, enhancing antioxidant capacity, and ameliorating organ dysfunction in various chronic nonspecific inflammatory conditions. The aim of this study was to evaluate the efficacy of PTE in IC/BPS and elucidate its underlying mechanisms using a rat model of cyclophosphamide (CYP)-induced interstitial cystitis. In comparison, chronic pain progression, histopathological features, and cytokine levels demonstrated that PTE mitigated the severity of symptoms in CYP-induced rats by inhibiting the NLRP3 inflammasome in a dose-dependent manner. Further mechanistic investigations indicated that PTE intervention alleviated oxidative stress in CYP-induced IC in rats via activation of the *Nrf2/HO-1* signaling pathway. Moreover, inhibitors of the *Nrf2/HO-1* pathway effectively blocked PTE-mediated attenuation of oxidative stress. The suppression of NLRP3 inflammasome activation by PTE could also be reversed by inhibition of the *Nrf2/HO-1* pathway. In vitro studies revealed that PTE enhanced the expression of nuclear factor erythroid 2-related factor 2 (Nrf2) and suppressed NLRP3 inflammasome activation in SV-HUC-1 cells exposed to lipopolysaccharide (LPS) and Adenosine Triphosphate (ATP). These findings collectively suggest that PTE treatment inhibits oxidative stress and suppresses NLRP3 inflammasome activation through modulation of the *Nrf2/HO-1* pathway.

## 1. Introduction

IC/BPS refers to a spectrum of conditions encompassing interstitial cystitis and painful bladder syndromes, characterized by chronic pelvic pain in the bladder region, urinary frequency, and urgency [1]. According to the 2014 guidelines from the American Urological Association, IC/BPS is defined as persistent or recurrent pelvic pain related to the bladder, in the absence of evident infection or other identifiable causes, accompanied by symptoms affecting both the upper and lower urinary tracts for at least six weeks [2]. The incidence of IC/BPS continues to increase, with recent statistics indicating approximately 653 females and 420 males per 100,000 individuals diagnosed with this condition [3]. Women are more susceptible to IC/BPS than men and often require treatment for recurrent symptoms, which can significantly impair physical and mental health. Additionally, due to the lack of specific pathological changes, there is no definitive consensus on IC/BPS treatment, and current clinical approaches primarily focus on symptom management [4]. While the precise etiology of IC/BPS remains elusive, accumulating evidence highlights the involvement of oxidative stress, infections, and immune dysregulation in its pathogenesis [5,6]. There are a large number of new therapies for IC/BPS, both pharmacological and nonpharmacological therapies, but their efficacy is uncertain. Therefore, further research is essential to elucidate the underlying mechanisms and develop effective treatments for IC/BPS.

PTE is a naturally occurring stilbene compound, mainly derived from rosewood, blueberry, grape, and other plants, with antioxidant, anti-inflammatory, anti-cancer, and other biological activities [7]. PTE effectively inhibits the generation of reactive oxygen species (ROS) and demonstrates potent scavenging activity against various free radicals, including DPPH, ABTS, hydroxyl radicals, superoxide anions, and hydrogen peroxide [8]. Beyond its ROS-scavenging capabilities, PTE also suppresses neutrophil activation, reduces inflammatory mediator production, and prevents endothelial cell apoptosis [9]. As a trans-stilbene compound and methylated derivative of resveratrol, PTE possesses enhanced bioavailability and stability compared to resveratrol, while retaining a wide range of biological activities, such as antioxidant [10], anti-tumor [11], lipid-lowering [12], and antibacterial effects [13]. Recent studies have highlighted the significant potential of PTE in mitigating chronic inflammatory diseases. Its anti-inflammatory efficacy has been confirmed in conditions such as ulcerative colitis [14], atopic dermatitis [15], periodontitis [16], and atherosclerosis [17]. These findings suggest that PTE may offer promising safety profiles and therapeutic potential for clinical applications. Nevertheless, the precise role of PTE in the pathogenesis and progression of IC/BPS requires further investigation.

The NLRP3 inflammasome complex consists of NOD-like receptor protein 3 (NLRP3), an apoptosis-associated speck-like protein containing a caspase recruitment domain (ASC), and caspase-1 [18]. This complex is closely associated with the progression of IC/BPS. The NLRP3 signaling pathway plays a pivotal role in the onset and development of bladder injury and inflammatory diseases [3]. The NLRP3 inflammasome complex formation leads to the cleavage of procaspase-1 into active caspase-1. Caspase-1 then processes the precursor forms of the inflammatory cytokines, pro-IL-1β and pro-IL-18, into their mature, active forms (IL-1β and IL-18). These mature cytokines are crucial mediators of inflammation and can result in tissue damage [19]. Recent studies have indicated that activation of the NLRP3 inflammasome exacerbates the inflammatory response and pain in bladder tissues of patients with IC/BPS [20]. Inhibition of NLRP3 inflammasome activation may thus represent a promising therapeutic strategy for IC/BPS. Furthermore, IC/BPS is attributed to an imbalance between peroxidation and antioxidation in the bladder [1]. ROS play a critical role in activating the NLRP3 inflammasome, leading to inflammation and tissue damage via mechanisms such as mitochondrial dysfunction, endoplasmic reticulum stress, and NF-κB signaling pathway activation [21]. In IC/BPS patients, high levels of oxidative stress can induce inflammation, resulting in adverse outcomes such as bladder pain and urinary dysfunction [22]. Therefore, antioxidant therapy targeting this imbalance could potentially serve as an effective treatment option for IC/BPS patients [23].

We report here that the effect of PTE on experimental models of interstitial cystitis was investigated in vivo and in vitro. The histological manifestations of the bladder were assessed, bladder pain was evaluated, and the expression levels of oxidative stress-related markers in plasma were measured following PTE treatment. Our study demonstrates that PTE can mitigate inflammation and bladder pain in rats with cyclophosphamide (CYP)-induced cystitis by inhibiting NLRP3 inflammasome activation and oxidative stress. Mechanistic investigation showed that the *Nrf2/HO-1* pathway plays an important role in the reduction of bladder tissue inflammation and oxidative stress by PTE. Our study supports the efficacy of PTE as a potential treatment for IC and provides an opportunity for developing novel therapeutic agents for clinical IC/BPS.

## 2. Results

### 2.1. Treatment with PTE Decreases Bladder Pain and the Inflammation of Bladder in CYP-Induced IC in Rats

**Figure 1:**
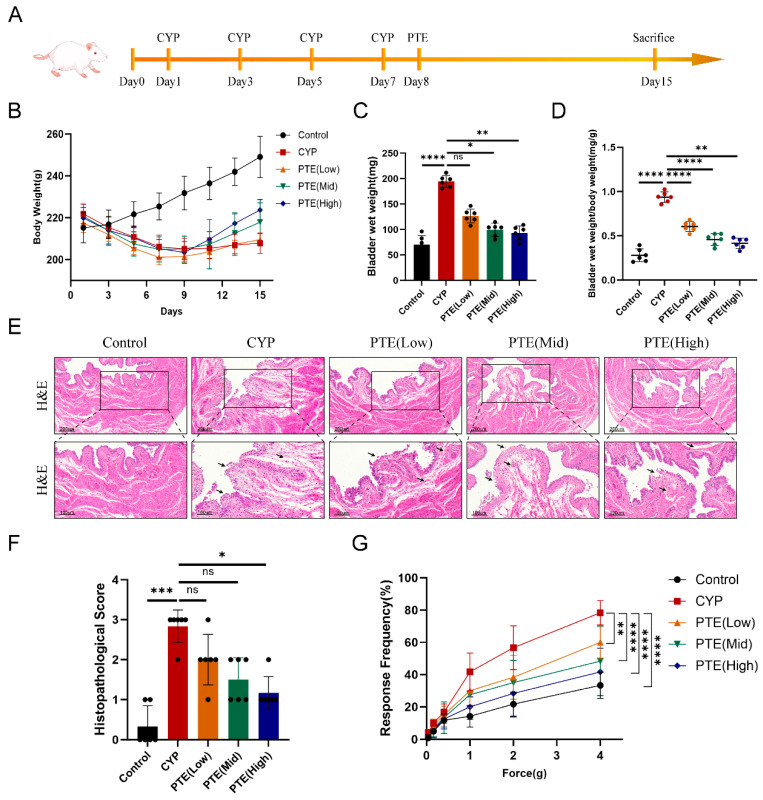
Pterostilbene (PTE) treatment ameliorates bladder inflammation and the pain response to mechanical stimulation. (**A**) The flow chart of the PTE treatment procedure. (**B**) The body weight of rats was evaluated in each group from 1 day to 15 day. (**C**) The bladder wet weight of rats was evaluated in each group. (**D**) Changes in bladder weight (mg) to body weight (g) ratio in the five groups. (**E**) Hematoxylin and eosin (H&E) staining showed that the infiltrating inflammatory cells in bladder tissues were significantly reduced in CYP-induced rats after PTE treatment. We use arrows to clearly indicate the separation and degeneration of the mucosal epithelium, as well as the edema. Scale bars represent 200 and 100 μM. (**F**) The Histopathological score of rats was evaluated in each group. (**G**) Pain response frequencies to mechanical stimulation of the rats in the five groups. The data are shown as the mean ± SD and were analyzed by Kruskal–Wallis non-parametric test (**F**), or two-way ANOVA analysis followed by Bonferroni post hoc test for multiple comparisons (**G**). “ns” indicates *p* > 0.05; * *p* < 0.05; ** *p* < 0.01; *** *p* < 0.001; **** *p* < 0.0001.

In this study, a rat model induced by CYP was utilized to evaluate the effects of PTE treatment on bladder inflammation and bladder pain associated with the inflammatory response (Figure 1A). As illustrated in Figure 1B, compared to the control group, a slight reduction in body weight was observed in the other groups from day 1 to day 15. Subsequently, we investigated the effects of PTE on the bladder. First, bladders were excised from the rats, and gross bladder specimens were examined. The results indicated that the bladder size (bladder wet weight, bladder wet weight/body weight ratio) decreased in a dose-dependent manner with increasing doses of PTE treatment (Figure 1C,D). H&E staining revealed that PTE treatment significantly reduced bladder inflammation, bleeding, and edema in CYP-induced rats in a dose-dependent manner (Figure 1E). Histological inflammation scores were also assessed, further confirming the reduction in inflammation as the concentration of PTE increased in rats treated with both CYP and PTE (Figure 1F). Patients with IC/BPS often experience symptoms such as urgency, frequent urination, nocturia, dysuria (burning sensation during urination), and dyspareunia, which have a serious negative impact on quality of life. Additionally, a significant proportion of patients report pelvic floor muscle pain (PFMP), which correlates with higher IC/BPS symptom scores and dyspareunia [4]. Therefore, the impact of PTE on bladder pain expression was explored, and the frequency of tactile allodynia responses in the PTE-treated group significantly and dose-dependently decreased compared to untreated CYP-induced rats. Taken together, these findings suggest that PTE treatment alleviates the severity and inflammatory pathology of CYP-induced IC in rats.

### 2.2. PTE Treatment Inhibits Activation of the NLRP3 Inflammasome

**Figure 2:**
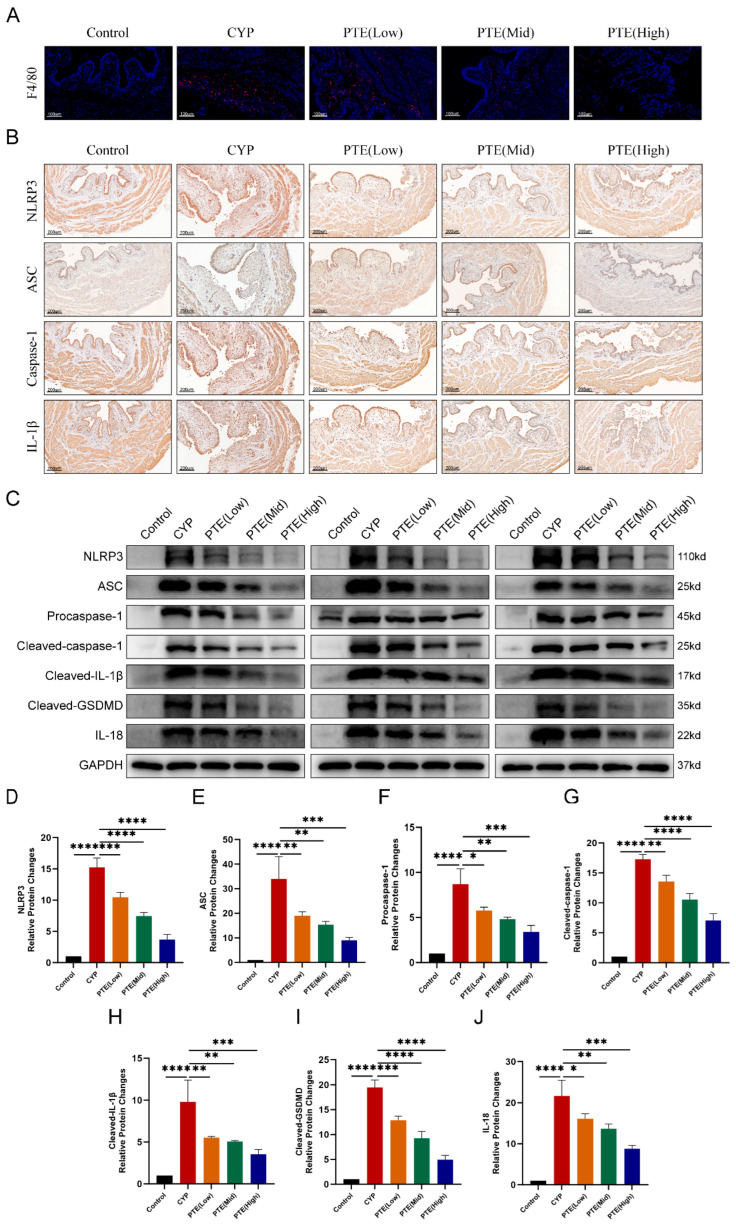
PTE treatment inhibits the activation of the NLRP3 inflammasome. (**A**) Representative images of immunofluorescence of F4/80+ macrophages in rat bladder treated with CYP and PTE. Scale bars represent 100 μM. (**B**) IHC analyses of NLRP3, ASC, caspase-1, and IL-1β expression in the bladder tissues of rats. Scale bars represent 200 μM. (**C**) Western blotting assay to measure the protein levels of NLRP3, ASC, Procaspase-1, Cleaved-caspase-1, Cleaved-IL-1β, Cleaved-GSDMD, and IL-18 in bladder tissues of CYP and PTE-treated rats. Statistical analysis of expression levels of NLRP3 (**D**), ASC (**E**), Procaspase-1 (**F**), Cleavedcaspase-1 (**G**), Cleaved-IL-1β (**H**), Cleaved-GSDMD (**I**), and IL-18 (**J**) detected by Western blotting assay. The data are shown as the mean ± SD and were analyzed by one-way ANOVA analysis followed by a Bonferroni post hoc test for multiple comparisons. * *p* < 0.05; ** *p* < 0.01; *** *p* < 0.001; **** *p* < 0.0001.

Previous studies have demonstrated a significant increase in mast cell infiltration and macrophage accumulation within the bladder tissue of IC/BPS patients [24]. This infiltration is closely associated with the initiation and progression of inflammatory responses, contributing to the characteristic bladder inflammation and associated pain symptoms observed in IC/BPS. In this study, immunofluorescence analysis was employed to evaluate changes in the proportion of macrophages in bladder tissue. The results revealed a marked increase in F4/80+ macrophage counts in the bladder tissue of the CYP group compared to the control group. Furthermore, PTE treatment significantly reduced F4/80+ macrophage infiltration in a dose-dependent manner in CYP-induced rats (Figure 2A). NLRP3 inflammasome activation plays a pivotal role in the development and progression of IC/BPS [3]. Thus, targeting this inflammasome may represent a promising therapeutic strategy. Immunohistochemical analysis showed that, compared to the control group, the CYP group exhibited elevated levels of NLRP3, ASC, cleaved-caspase-1, and IL-1β in bladder tissue (Figure 2B). With increasing doses of PTE treatment, the levels of NLRP3 inflammasome-related proteins in bladder tissue progressively decreased. Western blotting was conducted to quantify the protein levels of NLRP3, ASC, cleaved-caspase-1, cleaved-IL-1β, cleaved-GSDMD, and IL-18 in CYP-induced rats treated with varying concentrations of PTE (Figure 2C). The findings indicated that, compared to the control group, the CYP group displayed higher levels of NLRP3 inflammasome-related proteins, including NLRP3, ASC, cleaved-caspase-1, IL-1β, cleaved-GSDMD, and IL-18. Conversely, PTE treatment resulted in a dose-dependent reduction in these protein levels (Figure 2D–J). These results suggest that PTE treatment effectively inhibits the activation of the NLRP3 inflammasome in CYP-induced rats.

### 2.3. PTE Activates the Nrf2/HO-1 Pathway and Alleviates Oxidative Stress in CYP-Induced Rats

**Figure 3:**
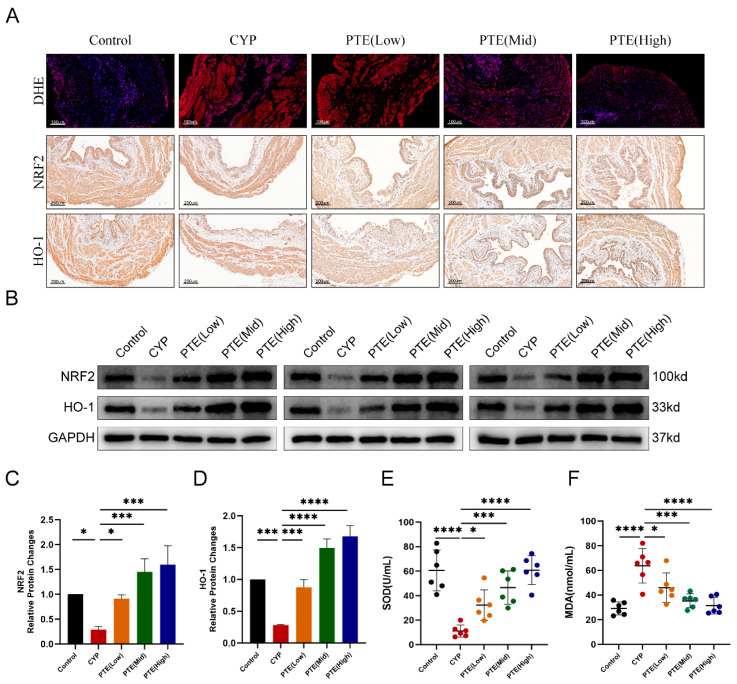
PTE inhibits oxidative stress and activates *Nrf2/HO-1* signaling in CYP-induced rats. (**A**) DHE staining to evaluate ROS production and IHC analyses of Nrf2 and HO-1 in bladder tissue of CYP and PTE-treated rats. Scale bars represent 100 μM. (**B**–**D**) Western blotting showing the protein levels of Nrf2 and HO-1 in bladder tissue of CYP and PTE-treated rats. (**E**,**F**) The levels of SOD and MDA in the serum of CYP and PTE-treated rats. The data are shown as the mean ± SD and were analyzed by one-way ANOVA analysis followed by Bonferroni post hoc test for multiple comparisons. * *p* < 0.05; *** *p* < 0.001; **** *p* < 0.0001.

Previous studies have demonstrated that oxidative stress is a critical mechanism underlying the pathogenesis of IC/BPS, characterized by oxidative stress at both local and systemic levels [5,25]. ROS produced during cell metabolism are abnormally elevated under conditions of oxidative stress, which is closely associated with inflammatory responses and cellular damage. In this study, dihydroethidium (DHE) staining was used to elucidate the role of ROS in CYP-induced rats, revealing an increase in ROS production in the CYP group. However, PTE treatment significantly reduced ROS production in a dose-dependent manner (Figure 3A). Nrf2, acting as a transcription factor, regulates the expression of key antioxidants such as HO-1 to decrease ROS levels [26]. Herein, the levels of Nrf2 and HO-1 were assessed using IHC and Western blotting analyses (Figure 3A,B). The results indicated a dose-dependent increase in Nrf2 and HO-1 levels following PTE treatment (Figure 3C,D). Superoxide dismutase (SOD), a marker of antioxidant capacity, catalyzes the conversion of superoxide radicals into harmless hydrogen peroxide and oxygen. Serum SOD levels were significantly decreased in the CYP group but were markedly elevated in a concentration-dependent manner following PTE treatment (Figure 3E). Malondialdehyde (MDA) levels serve as an indicator of oxidative membrane damage. Elevated serum MDA levels in the CYP group were reversed by PTE treatment (Figure 3F). These findings suggest that PTE alleviates oxidative stress in CYP-induced rats by activating the *Nrf2/HO-1* axis. Furthermore, PTE may suppress the NLRP3 inflammasome via this signaling pathway, thereby reducing inflammation in CYP-induced rats.

### 2.4. Blockade of Nrf2/HO-1 Signaling Abolished the PTE-Mediated Inhibition of Oxidative Stress

**Figure 4:**
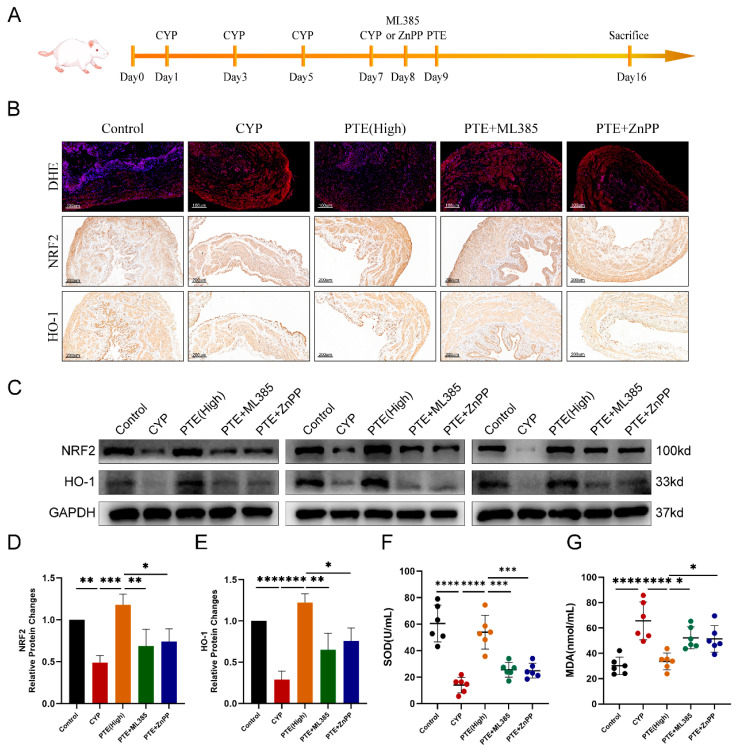
Pretreatment with ML385 or ZnPP reversed the inhibitory effects of PTE treatment on oxidative stress in rats. (**A**) The flowchart of the PTE, ML385 or ZnPP treatment procedures. (**B**) DHE staining to evaluate ROS production and IHC analyses of Nrf2 and HO-1 in bladder tissue of rats subjected to different treatments. Scale bars represent 100μM and 200 μM. (**C**–**E**) Western blotting assay to measure the protein levels of Nrf2 and HO-1 in bladder tissue of rats subjected to different treatments. (**F**,**G**) The expression levels of SOD and MDA in the serum of rats subjected to different treatments. The data are shown as the mean ± SD and were analyzed by one-way ANOVA analysis followed by a Bonferroni post hoc test for multiple comparisons. * *p* < 0.05; ** *p* < 0.01; *** *p* < 0.001; **** *p* < 0.0001.

To investigate whether the *Nrf2/HO-1* pathway mediates the inhibitory effect of PTE on oxidative stress, we administered the Nrf2 inhibitor ML385 (30 mg/kg), or the HO-1 inhibitor zinc protoporphyrin (ZnPP) (5 mg/kg), intraperitoneally to rats one day prior to PTE treatment. This intervention effectively suppressed Nrf2 and HO-1 activities, respectively. Detailed treatment protocols for PTE, ML385, and ZnPP are outlined in Figure 4A. DHE staining showed that both ML385 and ZnPP significantly abolished the inhibitory effect of PTE on ROS production (Figure 4B). The suppression of PTE-mediated Nrf2 transcriptional activity by ML385 was confirmed through IHC and Western blot analyses (Figure 4B–D). Additionally, ZnPP inhibited HO-1 activity, and pretreatment with ZnPP counteracted the stimulatory effect of PTE on HO-1 expression (Figure 4B,C,E). In the CYP group, serum SOD levels were significantly lower compared to the control rats, while serum MDA levels were markedly higher (Figure 4F,G). PTE treatment increased SOD levels and decreased MDA levels. However, pretreatment with either ML385 or ZnPP effectively antagonized these effects of PTE on SOD and MDA levels (Figure 4F,G). Collectively, these findings indicate that PTE mitigates oxidative stress via activation of the Nrf2/HO-1 pathway and that inhibition of this pathway abolishes the protective effect of PTE against oxidative stress.

### 2.5. Nrf2/HO-1 Signaling Blockade Reduces PTE-Mediated SUPPRESSION of Inflammasome Activation

**Figure 5:**
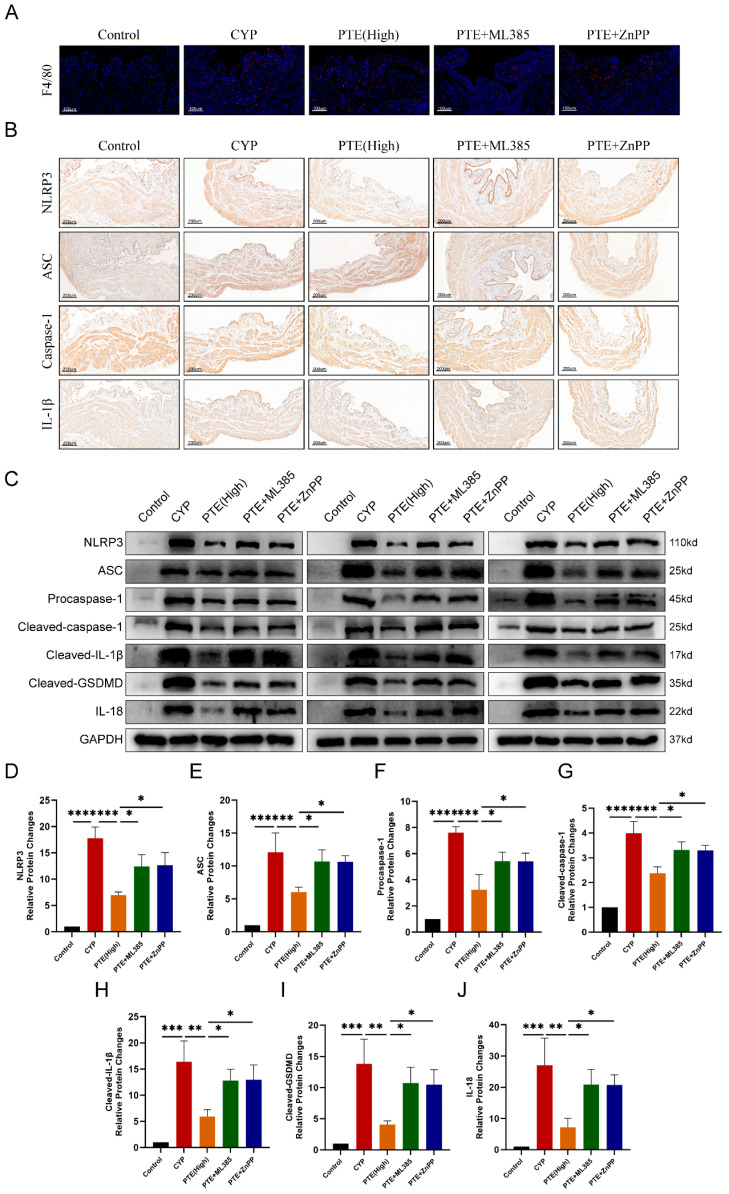
Pretreatment with ML385 or ZnPP reversed the inhibitory effects of PTE treatment on inflammasome activation in rats. (**A**) Representative images of immunofluorescence of F4/80+ macrophages in rat bladder treated with CYP and PTE. Scale bars represent 100 μM. (**B**) IHC analyses of NLRP3, ASC, Caspase-1, and IL-1β in bladder tissue of rats subjected to different treatments. Scale bars represent 200 μM. (**C**) Western blotting assay to measure the protein levels of NLRP3, ASC, Procaspase-1, Cleaved-caspase-1, Cleaved-IL-1β, Cleaved-GSDMD, and IL-18 in bladder tissues of CYP and PTE-treated rats. Statistical analysis of expression levels of NLRP3 (**D**), ASC (**E**), Procaspase-1 (**F**), Cleavedcaspase-1 (**G**), Cleaved-IL-1β (**H**), Cleaved-GSDMD (**I**), and IL-18 (**J**) detected by Western blotting assay. The data are shown as the mean ± SD and were analyzed by one-way ANOVA analysis followed by a Bonferroni post hoc test for multiple comparisons. * *p* < 0.05; ** *p* < 0.01; *** *p* < 0.001; **** *p* < 0.0001.

To explore whether the *Nrf2/HO-1* axis is involved in PTE-mediated inflammasome inhibition, an immunofluorescence analysis was performed to evaluate changes in the proportion of macrophages in bladder tissue. The results demonstrated that the number of F4/80+ macrophages was significantly higher in the ML385- and ZnPP-treated groups compared to the PTE-treated group (Figure 5A). Additionally, the expression levels of NLRP3, ASC, caspase-1, and IL-1β were assessed in the bladder tissue of CYP-induced rats following pretreatment with ML385 or ZnPP (Figure 5B). To further evaluate the involvement of the *Nrf2/HO-1* pathway in PTE-mediated inflammasome inhibition, we analyzed the protein levels of NLRP3, ASC, caspase-1, IL-1β, Cleaved-GSDMD, and IL-18 in the bladder tissues of CYP-induced rats (Figure 5C). Western blotting confirmed that the reductions in these proteins observed in the PTE-treated group were mitigated by pretreatment with ML385 or ZnPP (Figure 5C–J). These findings indicate that PTE inhibits inflammasome activation through the activation of the *Nrf2/HO-1* pathway, and blocking this pathway abolishes PTE’s inhibitory effect on inflammasome activation.

### 2.6. Suppression of Nrf2/HO-1 Blocks PTE-Mediated Reduction of Inflammation Activation in the CYP-Induced Rats

**Figure 6:**
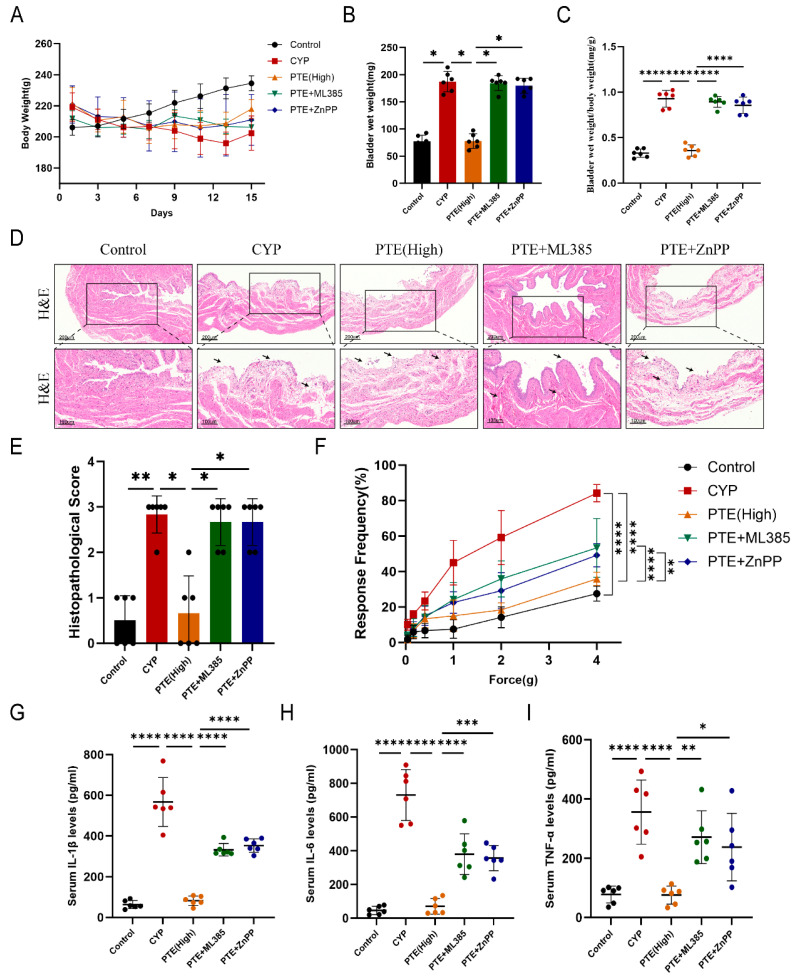
Pretreatment with ML385 or ZnPP reversed the anti-inflammatory effect of PTE. (**A**) The body weight of rats was evaluated in each group from 1 day to 15 days. (**B**) The bladder wet weight of rats was evaluated in each group. (**C**) Changes in bladder weight (mg) to body weight (g) ratio in the five groups. (**D**) H&E staining showed increased inflammatory cell infiltration in the bladder tissue of rats pretreated with ML385 or ZnPP compared to PTE-treated rats. We use arrows to clearly indicate the separation and degeneration of the mucosal epithelium, as well as the edema. Scale bars represent 200 and 100 μM. (**E**) Histopathological scores of rats after different treatments. (**F**) Pain response test results of rats subjected to different treatments. (**G**–**I**) Enzyme-linked immunosorbent assays (ELISAs) were performed to measure the IL-1β, IL-6, and TNF-α levels in the serum of rats subjected to different treatments. The data are shown as the mean ± SD and were analyzed by a Kruskal–Wallis non-parametric test (**E**), or two-way ANOVA analysis (**F**), or one-way ANOVA analysis followed by a Bonferroni post hoc test for multiple comparisons (**G**–**I**). * *p* < 0.05; ** *p* < 0.01; *** *p* < 0.001; **** *p* < 0.0001.

The effects of ML385 and ZnPP on PTE-mediated reductions in inflammation in CYP-induced rats were evaluated. In this study, the body weight and bladder wet weight of each rat in all groups were measured (Figure 6A,B). Compared to the ML385 and ZnPP pretreatment groups, there was a slight increase in body weight in the PTE (High) group from day 1 to day 15 (Figure 6A). Additionally, the bladder size, as indicated by the bladder wet weight and the ratio of bladder wet weight to body weight, exhibited a decreasing trend in the PTE (High) group (Figure 6B,C). H&E staining revealed that PTE reduced inflammatory cell infiltration, bleeding, and edema in the bladders of CYP-induced rats. Conversely, pretreatment with ML385 or ZnPP exacerbated this infiltration upon exposure to PTE (Figure 6D). Compared to the PTE (High) group, rats pretreated with ML385 or ZnPP demonstrated a significant increase in histopathological scores (Figure 6E). Furthermore, the tactile allodynia response rate was higher in rats pretreated with ML385 or ZnPP than in CYP-induced rats treated with PTE alone (Figure 6F). Moreover, the ELISA results indicated that serum levels of the inflammatory cytokines IL-1β, IL-6, and TNF-α were significantly elevated in the CYP group compared to the control group. PTE treatment effectively reduced these cytokine levels. However, pretreating CYP-induced rats with ML385 or ZnPP prior to PTE administration led to increased serum levels of IL-1β, IL-6, and TNF-α (Figure 6G–I). These findings suggest that PTE suppresses bladder inflammation via activation of the *Nrf2/HO-1* pathway, and blocking this pathway abolishes PTE’s anti-inflammatory effects in CYP-induced rats.

### 2.7. PTE Blocks NLRP3 Inflammasome Activation in SV-HUC-1 Cells by Activating Nrf2

**Figure 7:**
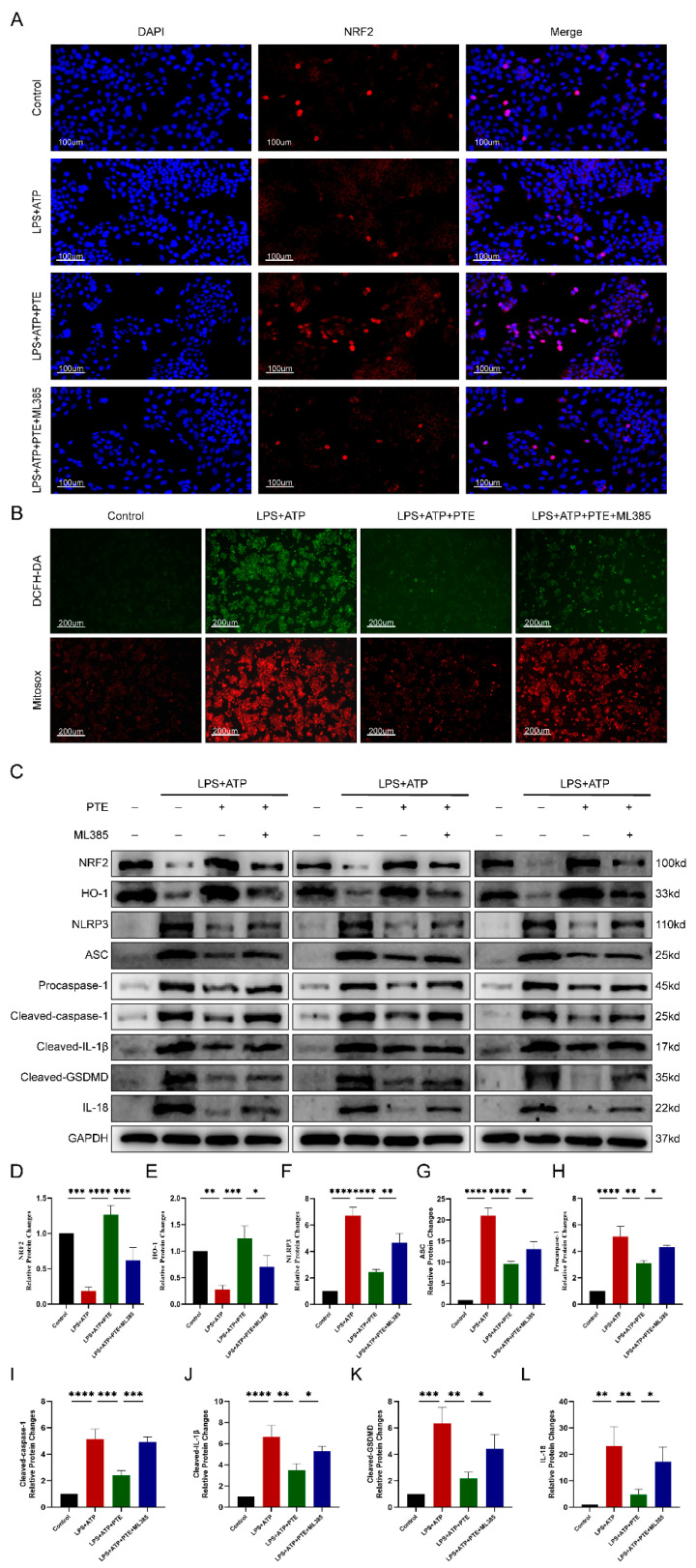
PTE activates the *Nrf2/HO-1* pathway to reduce oxidative stress levels and NLRP3 inflammasome activation in SV-HUC-1 cells. (**A**) Intracellular Nrf2 levels in SV-HUC-1 cells after different treatments. Cells were stained with DAPI and Nrf2. Scale bars represent 100 μM. (**B**) Intracellular and mitochondrial ROS levels in SV-HUC-1 cells after different treatments. Scale bars represent 200 μM. (**C**) Western blotting assay to measure the protein levels of Nrf2, HO-1, NLRP3, ASC, Procaspase-1, Cleaved-caspase-1, Cleaved-IL-1β, Cleaved-GSDMD, and IL-18 in cells. Statistical analysis of expression levels of Nrf2 (**D**), HO-1 (**E**), NLRP3 (**F**), ASC (**G**), Procaspase-1 (**H**), Cleavedcaspase-1 (**I**), Cleaved-IL-1β (**J**), Cleaved-GSDMD (**K**), and IL-18 (**L**) detected by Western blotting assay. The data are shown as the mean ± SD and were analyzed by one-way ANOVA analysis followed by a Bonferroni post hoc test for multiple comparisons. * *p* < 0.05; ** *p* < 0.01; *** *p* < 0.001; **** *p* < 0.0001.

To investigate whether PTE inhibits the NLRP3 inflammasome by activating Nrf2, SV-HUC-1 cells were treated with the Nrf2 inhibitor ML385, and intracellular Nrf2 levels were assessed via immunofluorescence analysis. Compared to control cells, the red fluorescence intensity in LPS + ATP-treated SV-HUC-1 cells was significantly reduced. Moreover, the red fluorescence in the PTE-treated group was markedly stronger than that in the LPS group, suggesting that PTE mitigated the reduction of Nrf2. Conversely, in the ML385-treated group, the red fluorescence intensity was weaker than in the PTE group, indicating that ML385 pretreatment effectively decreased intracellular Nrf2 levels (Figure 7A). The intracellular and mitochondrial ROS levels were, respectively, detected by DCFH-DA and Mitosox fluorescent probes (Figure 7B). The green and red fluorescence in the LPS + ATP-treated SV-HUC-1 cells increased relative to that in the control cells. In addition, the green and red fluorescence in the PTE-treated group was weaker than that in the LPS + ATP group, indicating that PTE prevented increases in OS. However, in the ML385 group, the green and red fluorescence was stronger than that in the PTE group, indicating that intracellular and mitochondrial ROS levels increased after pretreatment with ML385, confirming that PTE reduced ROS production through the Nrf2 pathway. Western blot analysis further demonstrated that PTE activated the *Nrf2/HO-1* signaling pathway and suppressed NLRP3 inflammasome activation. Additionally, the therapeutic effects of PTE were abrogated upon treatment with the Nrf2 inhibitor ML385 (Figure 7C–L).

## 3. Discussion

**Figure 8:**
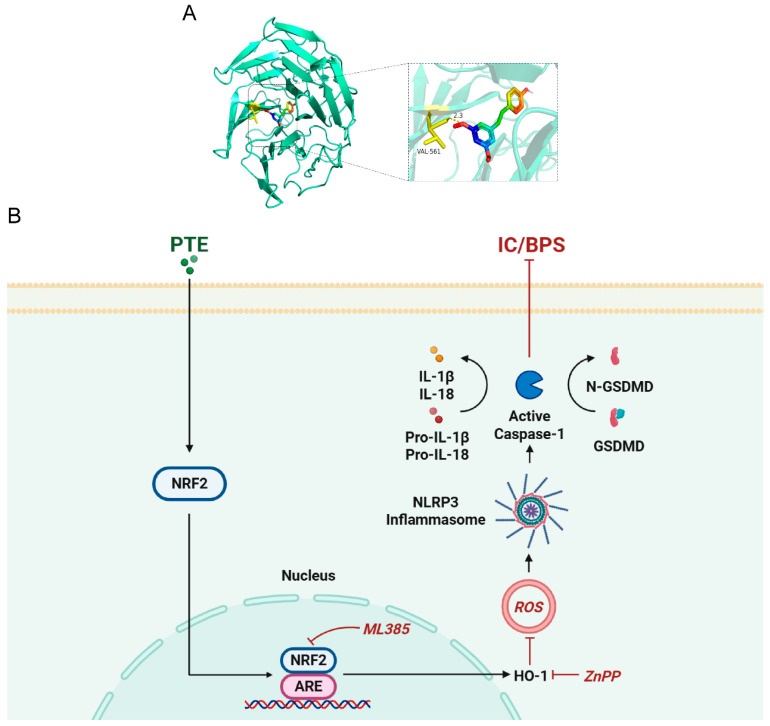
(**A**) Computational docking molecular simulation (the figure shows the 3D structure of the Nrf2 protein (green) and the binding sites of PTE to the Nrf2 protein (dashed box)). (**B**) Schematic diagram of the therapeutic effect of PTE in CYP-induced IC in rats and the associated mechanism. PTE treatment alleviated bladder inflammation and pain in CYP-induced IC rats by activating *Nrf2/HO-1* signaling to inhibit oxidative stress and NLRP3 inflammasome activation.

PTE demonstrates promising safety and clinical potential, as evidenced by its anti-inflammatory effects in various animal models of inflammatory diseases. However, the protective effects of PTE against IC/BPS via the Nrf2/NLRP3 axis have not been previously investigated. Our findings reveal that PTE significantly alleviates bladder pain and inflammation in CYP-induced rats, with therapeutic efficacy increasing in a dose-dependent manner. This suggests that PTE may possess therapeutic potential for the treatment of IC/BPS. Furthermore, PTE treatment effectively suppresses oxidative stress and NLRP3 inflammasome activation, leading to reduced ROS production and decreased levels of inflammatory cytokines, including IL-1β, IL-6, and TNF-α. Mechanistic studies indicate that the inhibitory effects of PTE on oxidative stress and NLRP3 inflammasome activation are mediated through *Nrf2/HO-1* signaling. The molecular docking results show that PTE binds to Nrf2 with a hydrogen bond to VAL-561 (distance ≈ 2.3 Å) and favorable hydrophobic interactions, suggesting a stable binding mode and potential modulation of Nrf2 activity (Figure 8A). When *Nrf2/HO-1* signaling is inhibited by ML385 or ZnPP, the anti-inflammatory properties of PTE in CYP-induced rats are abolished. Additionally, in vitro experiments confirm that PTE treatment enhances Nrf2 expression in LPS + ATP-stimulated SV-HUC-1 cells, while blocking the Nrf2 signaling pathway nullifies the therapeutic effects of PTE.

IC/BPS patients frequently experience inconvenience and embarrassment in social activities due to symptoms of frequent urination and pain, which are often accompanied by anxiety, depression, and other emotional issues. These psychological factors can exacerbate physical symptoms and complicate treatment regimens [27]. IC/BPS can be categorized into Hunner type (HIC/BPS) and non-Hunner type (NHIC/BPS) based on the presence or absence of Hunner lesions [28]. A histopathological analysis of bladder biopsies from IC/BPS patients reveals chronic inflammation characterized by infiltration of mast cells, macrophages, lymphocytes, and plasma cells [29]. Current treatments for IC/BPS include conservative management, pharmacotherapy, intravesical instillation, physical therapy, surgical intervention, and emerging therapies, all of which are aimed at alleviating symptoms and improving quality of life. However, despite personalized treatment plans tailored to individual patient conditions, these interventions have yet to yield consistently satisfactory clinical outcomes [30]. Previous studies have highlighted the multifaceted therapeutic potential of PTE, including its ability to ameliorate organ dysfunction, enhance antioxidant defenses, potentiate antitumor efficacy, and mitigate inflammatory responses [31,32]. PTE has demonstrated therapeutic benefits in various inflammatory diseases, such as neuroinflammation [33], allergic airway inflammation [34], hepatitis [32], osteoarthritis [35], and colitis [36]. In this study, we provide the first evidence that PTE treatment alleviates both bladder pain and inflammation in CYP-induced rats through its anti-inflammatory effects. Elevated levels of pro-inflammatory cytokines, including IL-6, IL-1β, and TNF-α, have been documented in both IC/BPS patients and CYP-induced rat models [24,37]. In this study, we demonstrated that PTE treatment significantly decreased the levels of cytokines, including IL-6, IL-1β, and TNF-α, in both the serum and bladder of rats induced by CYP, thereby suggesting its potential therapeutic efficacy for IC/BPS.

ROS plays a critical role in cell signaling, immune responses, and apoptosis. However, excessive ROS can induce oxidative stress and is closely linked to inflammatory diseases, cardiovascular disorders, and neurodegenerative conditions [38]. By modulating the expression of antioxidant genes, Nrf2 mitigates ROS production and its toxic effects, thereby safeguarding cells from oxidative stress-induced damage [39,40]. Specifically, elevated ROS levels trigger Nrf2 nuclear translocation, facilitating its specific binding to cis-regulatory antioxidant response element (ARE) motifs within target gene promoters [41]. Consequently, activating Nrf2 represents a promising therapeutic strategy for alleviating inflammation and oxidative stress-related damage. ROS exacerbates bladder inflammation and injury by inducing oxidative stress, activating inflammatory signaling pathways, compromising bladder epithelial barrier function, and intensifying neurogenic inflammation, thereby worsening symptoms in IC/BPS patients [24]. Despite ongoing research, the precise pathogenesis of IC/BPS remains elusive. Persistent inflammation in affected bladder tissues may generate ROS, potentially inducing significant alterations in protein structure and function, as well as DNA modifications [42]. D’Amico et al. demonstrated that Hidrox attenuates oxidative stress and inflammation associated with IC/BPS by enhancing Nrf2 signaling. Hidrox is an aqueous compound extracted from olive pulp and it contains 40–50% hydroxytyrosol, a natural phenolic compound known for its potent antioxidant and anti-inflammatory properties [43]. HO-1, an antioxidant enzyme regulated by Nrf2, catalyzes heme degradation to produce carbon monoxide, a metabolite with anti-inflammatory, anti-apoptotic, and vasodilatory properties, offering broad-spectrum tissue protection [44]. In this study, PTE treatment increased the expression of the antioxidant enzymes HO-1 and SOD, decreased MDA expression, inhibited the activation of the NLRP3 inflammasome, and attenuated inflammation and bladder pain in CYP-induced rats. PTE treatment alleviated oxidative stress in the bladder tissue of CYP-induced rats, boosted antioxidant capacity, and suppressed oxidative stress-related damage.

Inflammasome is a multi-protein complex within the innate immune system that plays an important role in detecting intracellular danger signals, such as PAMPs or DAMPs, and initiating inflammatory responses [45]. In CYP-induced rats, NLRP3 inflammasome activation triggers a cascade of inflammatory responses within the bladder, exacerbating inflammation and increasing pain levels [46]. Studies have shown that elevated levels of IL-1β and IL-18 are closely associated with the severity of IC/BPS. These cytokines are significantly upregulated in both the bladder tissue and serum of IC/BPS patients, indicating their pivotal roles in the disease onset and progression [47,48]. Therefore, targeted inhibition of NLRP3 inflammasome signaling may represent a promising therapeutic strategy for managing IC/BPS. This study revealed a markedly increased expression of NLRP3 inflammasome-related proteins in CYP-induced rats, suggesting robust NLRP3 inflammasome activation. Treatment with PTE effectively attenuated this activation.

Additionally, we also conducted validation of the in vitro treatment effects of PTE, yielding consistent findings. Cellular antioxidant responses are predominantly regulated by the *Nrf2/HO-1* signaling pathway, which plays a critical role in maintaining redox homeostasis and protecting against oxidative stress-induced damage. In response to excessive oxidative stress, Nrf2 translocates to the nucleus, where it binds to AREs in the DNA. This interaction triggers the transcriptional activation of downstream genes, such as HO-1, thereby initiating a protective antioxidant response to counteract oxidative damage [41]. The present study indicates that PTE enhances antioxidant capacity and reduces ROS formation through modulation of the *Nrf2/HO-1* pathway [34]. In our investigation, PTE treatment was found to upregulate the levels of the antioxidant HO-1 by activating the Nrf2 pathway, further regulating ROS generation. On the one hand, when intracellular ROS levels increase, ROS can directly oxidatively modify the NLRP3 protein, altering its conformation and promoting its oligomerization and activation [49]. On the other hand, ROS can also indirectly activate the NLRP3 inflammasome by influencing upstream signaling pathways of NLRP3 or by modulating mitochondrial function to affect NLRP3 activation [21]. Studies have shown that factors such as mitochondrial ROS and K+ efflux are upstream triggering factors that link mitochondrial oxidative stress to the activation of the NLRP3 inflammasome [50]. The activation of NLRP3 is accompanied by a significant accumulation of mitochondrial superoxide mtROS, which is consistent with the previously reported core role of mtROS as the “second signal” for NLRP3 [51]. mtROS can also enhance the sensitivity of the purinergic receptor P2X7R and induce K+ efflux, which is also the most universal trigger factor for NLRP3 [52]. Given that mtROS can induce the opening of K+ channels, there may be a synergistic effect between them. We investigated whether the inhibitory effect of PTE on NLRP3 inflammasome activation is mediated through the *Nrf2/HO-1* pathway by promoting ROS clearance. Our results demonstrated that rats treated with PTE exhibited increased levels of Nrf2, along with markedly reduced levels of NLRP3 inflammasome-related proteins, such as IL-1β and cleaved-GSDMD. This indicates that the *Nrf2/HO-1* pathway may play a pivotal role in the anti-inflammatory mechanism of PTE by modulating intracellular ROS levels, thereby suppressing NLRP3 inflammasome activation. Our study also indicated that, under conditions of NLRP3 activation, there was a surge in mtROS, and the total ROS levels rose correspondingly. Furthermore, in PTE-treated rats, oxidative stress, NLRP3 inflammasome activation, and anti-inflammatory effects were abolished upon blocking the *Nrf2/HO-1* signaling pathway using ML385 and ZnPP. Nevertheless, our study has certain limitations that require further investigation. While PTE exerts anti-inflammatory effects via multiple signaling pathways, additional downstream pathways remain to be identified to provide a more comprehensive understanding of its mechanisms. Moreover, pharmacological inhibition using ML385 and ZnPP may exhibit off-target effects. To address this limitation and further validate the role of the *Nrf2/HO-1* pathway, future studies will utilize *Nrf2/HO-1* knockout rats. This approach will facilitate a more rigorous examination of the underlying mechanisms and help confirm the specificity of the observed effects.

## 4. Materials and Methods

### 4.1. Animals

The healthy female Sprague-Dawley (SD) rats used in this study were purchased from the Beijing Charles River Laboratory Animal Technology Co., Ltd., Beijing, China. The rats were 6 and 8 weeks old and housed in the Institute of Zoology Chinese Academy of Sciences under pathogen-free conditions, with free access to water and food, and they were switched between light and dark cycles every 12 h. The animal room has controlled temperature (18 °C–23 °C) and humidity (40–60%). Female rats were randomly divided into different groups as specified. Rats were euthanized by CO2 inhalation following AVMA and institutional guidelines. Tissues were immediately snap-frozen and stored at −80 °C, or formalin-fixed for subsequent histological evaluation. All animal procedures were conducted in accordance with protocols approved by the Ethics Committee of Laboratory Animal Welfare, Institute of Animal Studies, Chinese Academy of Sciences (Approval No. IOZ-IACUC-2024-178).

### 4.2. Reagents

The manufacturer information for the reagents and antibodies used in the experiment is summarized as follows. PTE (GN10244), ML385 (GC19254), ZnPP (GC11208), lipopolysaccharide (LPS) (L2880), adenosine triphosphate (ATP) (11140965001), and ROS staining solution (GD20803) were purchased from GlpBio Technology Inc. (Montclair, CA, USA). Cyclophosphamide (CYP) (C0768) was obtained from Sigma-Aldrich (St. Louis, MO, USA). Oxidative stress test kits, including MDA (02.13065) and SOD (02.13067) test kits, were acquired from Eallbio (Beijing, China). ELISA reagents for quantifying IL-1β (RK00009), IL-6 (RK00020), and TNF-α (RK00029) were supplied by Abclonal (Wuhan, China). Primary antibodies included anti-Nrf2 (16396-1-AP; ProteinTech, Rosemont, IL, USA), anti-HO-1 (10701-1-AP; ProteinTech), anti-NLRP3 (DF7438; Affinity Biosciences, Cincinnati, OH, USA), anti-ASC (DF6304; Affinity Biosciences), anti-Caspase-1 (22915-1-AP; ProteinTech), anti-IL-1β (AF5103; Affinity Biosciences), anti-GSDMD (AF4012; Affinity Biosciences), anti-IL-18 (DF6252; Affinity Biosciences), and anti-GAPDH (#5174; CST, Danvers, MA, USA). The secondary antibody used was horseradish peroxidase-conjugated goat anti-rabbit (SA00001-2; ProteinTech).

### 4.3. Animal Treatment

As previously described, to induce experimental IC/BPS in rats, CYP (75 mg/kg body weight) was administered via intraperitoneal injection on alternate days (days 1, 3, 5, and 7), while the control group received saline on the same schedule [53,54]. The success of the model was assessed using HE pathology scores, serum inflammatory factor levels, and tactile pain responses. PTE treatment commenced on day 2 following modeling and continued for one week. CYP and PTE powder were dissolved in 0.9% saline. ML385 powder was dissolved in 100% DMSO to make a stock solution, diluted into a usable solution with a mixture (5% DMSO, 40% PEG300, 5% Tween 80, and 50% sterile water). In the dark, ZnPP powder was dissolved in 100% DMSO to make a stock solution, diluted into a usable solution with a mixture (5% DMSO, 40% PEG300, 5% Tween 80, and 50% sterile water). The experiment was conducted in two phases. In the first phase, SD rats were randomly assigned to the following five groups: the control group, the CYP group (model with saline gavage), the low-dose PTE group (model with PTE 10 mg/kg gavage), the medium-dose PTE group (model with PTE 20 mg/kg gavage), and the high-dose PTE group (model with PTE 40 mg/kg gavage) (*n* = 6), totaling 30 female SD rats. Each group received oral administration according to the corresponding regimen. In the second phase, SD rats were randomly divided into the following five groups: the control group, the CYP group (model with saline gavage), the CYP group treated with high-dose PTE (PTE 40 mg/kg gavage) and ML385 (30 mg/kg), and the CYP group treated with high-dose PTE (PTE 40 mg/kg gavage) and ZnPP (5 mg/kg) (*n* = 6), totaling 30 female SD rats. One day prior to PTE gavage, rats received intraperitoneal injections of ML385 or ZnPP, while the control group received vehicle instead [55].

### 4.4. Cell Culture and Experimentation

SV-HUC-1 cells used in this study were obtained from Eallbio (Beijing, China). SV-HUC-1 cells were cultured at 37 °C in a 5% CO2 incubator using DMEM/F-12K medium (31800-105, Gibco, Waltham, MA, USA) supplemented with 10% fetal calf serum (FBS; 1009141C, Gibco, USA), 100 U/mL penicillin, and 100 µg/mL streptomycin (15140122, Gibco, USA). The cells were divided into the following four groups: control; LPS + ATP; LPS + ATP + 40 µmol/L PTE; and LPS + ATP + 40 µmol/L PTE + 10 µmol/L ML385. After rinsing with PBS, the cells were incubated with 40 µmol/L PTE in serum-free DMEM/F-12K for 4 h, followed by a second wash with PBS and subsequent incubation with LPS (1 µg/mL) + ATP (2.5 mM) in serum-free DMEM/F-12K for 12 h. In the ML385-treated group, 10 µmol/L ML385 was added to the serum-free DMEM/F-12K prior to PTE treatment.

### 4.5. Behavioral Testing

Before being sacrificed, rats in different groups were assessed for tactile allodynia in the lower abdominal region near the bladder. As previously described [56], behavioral tests were conducted using von Frey filaments in plastic chambers equipped with a wire grid floor that isolated the surface from the surrounding environment. The rats were given 30 min to acclimate to the new environment before testing commenced. Subsequently, tactile allodynia and hyperalgesia were evaluated in each rat using von Frey filaments with forces of 0.04, 0.16, 0.4, 1.0, and 4.0 g. During the experiment, the filaments were applied 20 times at intervals of one to two seconds, with a two-minute rest period between applications to avoid the “wind-up” phenomenon. Different areas within the region were stimulated sequentially to minimize habituation or sensitization effects. Positive responses to filament stimulation were defined as follows: (1) sharp retraction of the abdomen; (2) immediate licking or scratching of the stimulated area; and (3) jumping. The results are expressed as the percentage of positive responses.

### 4.6. Measurement of Bodyweight, Bladder Wet Weight and Histologic Evaluation of Bladder Tissue

The body weight and bladder wet weight of each rat in each group were measured by a precision balance (Sartorius, Göttingen, Germany). During the experiment, bladder tissues were collected from rats, fixed in paraformaldehyde for 24 h, dehydrated using a graded alcohol series and xylene, and subsequently embedded in paraffin wax. The tissues were then sectioned into 4-μM-thick slices for further analysis. H&E staining was applied to assess inflammation in the bladder tissues. Following the methods described in previous studies [57], the degree of inflammation was quantified on a 6-point scale from 0 to 5. Possible outcomes were as follows: 0, No significant inflammation or epithelial changes; (1) Minimal inflammation in the lamina propria, no muscularis propria involvement, edema, hemorrhage, or urothelial changes; (2) Mild inflammation in the lamina propria with scattered cells, mild edema, or hemorrhage, no muscularis propria inflammation or significant urothelial changes; (3) Mild to moderate inflammation in the lamina propria, focal extension into the muscularis propria; (4) Moderate inflammation with frequent cells in both layers; (5) Severe inflammation in both layers with significant findings like ulceration, edema, hemorrhage, and fibrin deposition.

### 4.7. Immunohistochemistri (IHC) Analysis

The paraffin-embedded tissue specimens were sectioned at a thickness of 4 μM. Subsequently, the sections underwent deparaffinization with xylene, rehydration through a graded alcohol series, and antigen retrieval via incubation in 0.01 M citric acid buffer (pH 6.0). Following this, the sections were treated with 3% hydrogen peroxide (Beijing Zhongshan Jinqiao Biotechnology Co., Ltd., Beijing, China) for 10 min at room temperature to inhibit endogenous peroxidase activity. The sections were then washed three times with PBS (pH 7.4), blocked with 10% bovine serum albumin for 1 h, and incubated overnight at 4 °C with primary antibodies. After additional washing with PBS (three times), the sections were incubated with horseradish peroxidase-conjugated goat anti-rabbit IgG (1:200) at 37 °C for 30 min. Finally, antibody complexes were visualized using DAB (Sigma-Aldrich, St. Louis, MO, USA) and counterstained with hematoxylin following PBS washes (3 × 5 min).

### 4.8. Immunofluorescence

The 4-μM-thick bladder tissue sections were sequentially dehydrated using ethanol solutions of 100%, 90%, 70%, and 50% concentrations, with each step lasting 5 min. Subsequently, the sections were incubated in a 3% hydrogen peroxide solution (provided by Beijing Zhongshan Jinqiao Biotechnology Co., Ltd.) at room temperature for 10 min to quench endogenous peroxidase activity. To enhance membrane permeability, the sections were treated with 0.5% Triton X-100 in PBS for 10 min. Nonspecific binding was minimized by blocking the sections with 10% bovine serum albumin (BSA) for 1 h at room temperature. The sections were then incubated overnight at 4 °C with the respective primary antibodies (F4/80 diluted at 1:500, Nrf2 diluted at 1:500) to facilitate specific antigen–antibody interactions. Following this, the sections were washed with PBS and incubated with DAPI and fluorescein-conjugated secondary antibodies for 1 h at room temperature. Finally, the sections were examined and imaged using a confocal laser-scanning microscope (Olympus Fluoview FV3000, Tokyo, Japan). For statistical analysis, six random fields were selected.

### 4.9. ROS Production Assay

The levels of ROS in bladder tissues were evaluated using DHE staining. Fresh bladder tissues were stored at −80 °C in an ultra-low-temperature freezer and subsequently sectioned into 5-μM-thick frozen slices with precision. The frozen sections were then incubated with DHE (10μM) for 1 h at room temperature in the dark. Following incubation, the sections were gently washed three times with PBS to remove unbound dye. Cell nuclei were counterstained with DAPI for 10 min at room temperature (25 °C) in the dark. In parallel, ROS levels in SV-HUC-1 cells were measured according to the manufacturer’s instructions using a commercial kit. SV-HUC-1 cells were incubated with DCFH-DA and Mitosox for 20 min at 37 °C and subsequently analyzed and imaged using an FV1000 Fluoview confocal laser-scanning microscope (Olympus, Tokyo, Japan).

### 4.10. Evaluation of Oxidative Stress

SOD is a marker reflecting the free radical scavenging capacity, while MDA indicates severe membrane damage and signifies free radical levels and oxidative stress [58]. The SOD and MDA levels in rat serum were measured using commercial kits according to the manufacturer’s instructions. Their concentrations were calculated based on these guidelines and normalized to the total protein concentration.

### 4.11. Western Blotting Assay

Proteins were extracted from rat bladder tissue, added to SV-HUC-1 cells, and lysed in RIPA buffer (Beyotime Biotech, Haimen, Jiangsu, China) supplemented with PMSF, phosphatase inhibitors, and protease inhibitors. The lysates were incubated for 20 min at 4 °C. Protein concentrations were determined using a BCA kit (Sigma-Aldrich, St. Louis, MO, USA) according to the manufacturer’s instructions. Protein samples from bladder tissues were denatured by boiling in sodium dodecyl sulfate polyacrylamide gel electrophoresis (SDS-PAGE) sample loading buffer. The denatured proteins were separated by SDS-PAGE on 10% gels and subsequently transferred onto nitrocellulose membranes using a semidry transfer system. Nonspecific binding was blocked by incubation with 5% (w/v) non-fat milk in Tris-buffered saline with Tween 20 (TBST). The membranes were then incubated overnight at 4 °C with primary antibodies against NLRP3 (1:1000), ASC (1:1000), Caspase-1 (1:1000), IL-1β (1:1000), GSDMD (1:1000), IL-18 (1:1000), Nrf2 (1:1000), and HO-1 (1:1000). After three washes with TBST, the membranes were incubated with horseradish peroxidase-conjugated secondary antibodies (1:5000) for 2 h at room temperature. Protein bands were visualized using an EZ-ECL Kit (Biological Industries, Kibbutz Beit Haemek, Israel) on a ChemiScope 5600 imaging system (Clinx Science Instruments, Shanghai, China), and band density was quantified using ImageJ software, version 6.0 (NIH, Bethesda, MD, USA).

### 4.12. Elisa

Cytokine levels of IL-1β (RK00009; Abclonal; Wuhan, China), IL-6 (RK00020; Abclonal; Wuhan, China), and TNF-α (RK00029; Abclonal; Wuhan, China) in rat serum were quantified using ELISA kits (Abclonal; Wuhan, China). All procedures were carried out strictly in accordance with the manufacturer’s instructions.

### 4.13. Data Analysis

All experiments were independently repeated at least three times to ensure the reliability and accuracy of the data. Statistical analyses were performed using a one-way ANOVA followed by a Bonferroni post hoc test for multiple group comparisons, a two-way ANOVA for analyzing two-factor experimental data, and a Kruskal–Wallis nonparametric test for comparing ranked data across multiple groups. All statistical analyses were conducted using GraphPad Prism version 6.0 (GraphPad Software, La Jolla, CA, USA). Data are expressed as the mean ± SD, with *p* < 0.05 considered statistically significant. In the figures, “ns” denotes *p* > 0.05; * *p* < 0.05; ** *p* < 0.01; *** *p* < 0.001; **** *p* < 0.0001.

## 5. Conclusion

In conclusion, we used Pterostilbene and explored its protective effect on interstitial cystitis. Our study initially revealed the therapeutic effects of PTE in CYP-induced rats, demonstrating that exogenous PTE supplementation significantly alleviated bladder pain and inflammation, thereby highlighting its potential as a promising therapeutic agent for IC/BPS. PTE treatment inhibited the activation of the NLRP3 inflammasome and oxidative stress by activating the *Nrf2/HO-1* signaling pathway (Figure 8B). This novel finding not only highlights the potential of PTE as a promising therapeutic agent for IC/BPS, but also offers fresh insights into its application in managing CYP-induced IC in rats.

## Data Availability

The data used to support the findings of this study are available from the corresponding author upon request.
